# Therapeutic Use of Low-Dose Local Anesthetics in Pain, Inflammation, and Other Clinical Conditions: A Systematic Scoping Review

**DOI:** 10.3390/jcm12237221

**Published:** 2023-11-21

**Authors:** David Vinyes, Montserrat Muñoz-Sellart, Lorenz Fischer

**Affiliations:** 1 Institute of Neural Therapy and Regulatory Medicine, 08202 Sabadell, Spain; montse@institutdeterapianeural.cat; 2 Master of Permanent Training in Medical and Dental Neural Therapy, University of Barcelona—IL3, 08018 Barcelona, Spain; 3 Neural Therapy Research Foundation, 08202 Sabadell, Spain; 4 Formerly Neural Therapy, Institute of Complementary and Integrative Medicine (IKIM), University of Bern, 3012 Bern, Switzerland; lorenz.fischer@unibe.ch

**Keywords:** local anesthetics, pain, neural therapy, therapeutic local anesthesia, procaine, lidocaine

## Abstract

The use of low-dose local anesthetics (LAs) has significantly transformed patient care by providing rapid and effective relief of pain and other clinical conditions while minimizing recovery time. This study aims to identify and describe the existing scientific evidence on the therapeutic use of low-dose LAs in various conditions and to identify gaps in the current literature in order to prioritize future research. This systematic scoping review adhered to the methodological guidelines outlined in the Arksey and O’Malley framework, which includes five distinct stages. Of the 129 studies included, 37.98% (*n* = 49) were clinical trials, 55.03% (*n* = 71) were observational studies, and 6.97% (*n* = 9) were systematic reviews. The most commonly reported indication for the use of low-dose LAs was chronic pain management (72.86%), followed by acute pain management (13.17%). Additionally, non-pain-related indications were also identified (13.95%). Overall, the administration of low-dose, short-acting LAs demonstrated favorable outcomes in terms of pain management and reduction in anxiety and depression scales, thereby having a positive impact on the patients’ quality of life. This review represents the first systematic scoping review regarding the therapeutic role of LAs. To substantiate the reported positive effects on efficacy and safety, further rigorous research comprising larger, well-designed randomized controlled trials (RCTs) and long-term outcome monitoring is imperative.

## 1. Introduction

The use of local anesthetics (LAs) for therapeutic purposes dates to their discovery when the Austrian ophthalmologist Carl Koller performed the first surgery using the anesthetic properties of cocaine in 1894 [1]. In 1905, Einhorn synthesized the local anesthetic procaine, and, a year later, Spiess reported the rapid reduction of inflammation with procaine injections, attributing this effect to its action on the nervous system [2]. Over the years, the medical community has expanded on the pioneering ideas of the Huneke brothers and others, leading to the widespread use of LAs for therapeutic purposes in Europe, often referred to as neural therapy (NT) or therapeutic local anesthesia [3,4,5,6,7,8].

The therapeutic use of LAs in the treatment of pain and various other clinical conditions has been well-documented [9,10,11,12] and accepted, as evidenced by its inclusion in the mandatory basic insurance in some countries, such as Switzerland, Austria, or Colombia. Traditionally used in the surgical setting for short-term analgesia, the therapeutic use of LAs now aims to provide sustained relief of pain and other dysfunctions by targeting the autonomic nervous system (ANS). The ANS plays a central role in the regulation of inflammatory and immune processes, (micro) circulation and thus pain, and other clinical conditions [13,14,15,16,17,18,19,20].

One of the intriguing aspects of this therapy is the use of low-dose, short-acting LAs in areas of injury or inflammation, nerves, ganglia, and others, such as myofascial trigger points. The underlying principle is to modulate the auto-regulatory mechanisms and plastic properties of the nervous system, particularly the ANS [4,5,17,18,21,22,23,24,25]. The role of the ANS in the modern understanding of inflammation and pain is fundamental, as ANS controls reflexive neuroimmunological and inflammatory cascades [13,16,17,26,27,28,29].

The global acceptance and application of LA therapy are rapidly expanding, and this review aims to contribute to the ongoing development of this therapeutic approach. We focus on studies using low-dose, short-acting LAs, without any additives or combinations with other drugs, at concentrations up to 2%, for therapeutic purposes, without the intent to achieve local anesthesia, based on the criteria known as NT [3,4,5]. The efficacy and safety of this approach have been explored in conditions ranging from pain, inflammation, and other conditions, such as arterial hypertension and post-traumatic stress syndrome to post-COVID-19 patients [3,9,10,11,17,21,22,30,31,32,33,34,35,36,37,38,39,40,41,42,43,44,45,46,47,48,49,50,51]. However, a comprehensive understanding of the scope and design of these studies is unknown. This review attempts to summarize and describe the current scientific evidence on therapeutic LAs for pain management and other conditions while also identifying gaps in the literature to guide future research.

## 2. Materials and Methods

### 2.1. Study Design

To gather relevant information, a systematic scoping review was conducted in accordance with the methodological guidelines of the Arksey and O’Malley framework, which recommends the following stages: (1) identifying the research question; (2) identifying relevant studies; (3) study selection; (4) charting the data; and (5) collating, summarizing, and reporting the results. The report of this scoping review was compiled by the Preferred Reporting Items for Systematic Reviews and Meta-Analyses (PRISMA) extension for scoping reviews (PRISMA-ScR) [52].

See Annex A for the PRISMA-ScR Checklist of the present study.

### 2.2. Identifying the Research Question

To develop our research question, we used the PICO (population, intervention, comparator, and outcomes) framework [53].

The research question was as follows: In patients with various medical conditions, what does the existing literature reveal about the therapeutic application of low-dose LAs with concentrations below 2%?

### 2.3. Identifying Relevant Studies

We selected all studies published in the literature that used LAs alone and included any measures of efficacy or safety (such as reporting toxicity) or outcomes with a therapeutic purpose. We included studies in which the intervention involved LAs, with or without a comparator. For studies that included a comparator, we only selected those that contained at least one group that used LAs alone or diluted with a saline solution. Studies that focused on other types of outcomes (such as cost-effectiveness and prognosis) were excluded.

Both experimental and quasi-experimental study designs were considered, including randomized controlled trials, non-randomized controlled trials, pre-post studies, and interrupted time-series studies. In addition, analytical observational studies, including prospective and retrospective cohort studies, case-control studies, and analytical cross-sectional studies, were considered for inclusion. This review also included descriptive observational study designs such as case series, individual case reports, descriptive cross-sectional studies, as well as qualitative studies, systematic reviews, and meta-analyses that met the inclusion criteria.

Conversely, we excluded narrative reviews, expert opinions, and letters to the editor. Studies involving systemic use or combination with other drugs, such as anti-inflammatory drugs, corticosteroids, or phytotherapy, as well as non-pharmacological interventions such as acupuncture, were also excluded.

Our goal was to identify all published studies on the use of low-dose Las for therapeutic purposes. Our literature search had no language or time constraints. To achieve this, we developed a search strategy based on the terms included in Annex B and applied it to the following databases (*n* = 4):

- MEDLINE via PubMed
- Cochrane Library—including the Cochrane Database of Systematic Reviews (CDSR) and CENTRAL (clinical trials)
- LILACS

We conducted a concise search on Google Scholar to identify potential studies that may not have been included in the selected databases. Additionally, we conducted a brief search by referencing the included studies.

### 2.4. Study Selection

Two reviewers independently assessed articles based on the inclusion criteria by title and abstract. After this initial screening stage, the full texts of the articles that met these criteria were obtained, and a second review was conducted to confirm inclusion based on the full text. For an article to proceed to this second screening phase, it had to meet all the inclusion criteria and none of the exclusion criteria. Any disagreements were resolved by discussion or consultation with a third reviewer. We used Rayyan Qatar Computing Research Institute (QCRI)—a web and mobile app for systematic reviews, 2016, as the data management software for both screening phases.

### 2.5. Charting the Data

We developed a data extraction table to collate the relevant data for this review and to facilitate discussion of the variables. The extraction variables included: author(s), year of publication, source of origin/country of origin, objectives, characteristics of the study population and sample size, type of study, type of intervention and comparator (if applicable), efficacy or safety outcome, how the outcome was measured, and key findings relating to the review question. Additionally, data regarding the safety of the intervention were specifically recorded in those articles where such information was provided.

Two reviewers independently extracted and verified the data, and any discrepancies were resolved by a third reviewer.

### 2.6. Collating, Summarizing, and Reporting the Results

Regarding the presentation of results, we performed a narrative synthesis in which we used the text and tables from the studies to provide a descriptive summary and explanation of the study characteristics and findings. We presented this information visually and in evidence tables. The characteristics and main findings of the included studies were presented in a narrative synthesis that highlighted the gaps in the evidence and provided potential interpretations and implications of the results.

## 3. Results

### 3.1. Search Results and Study Selection

**Figure 1:**
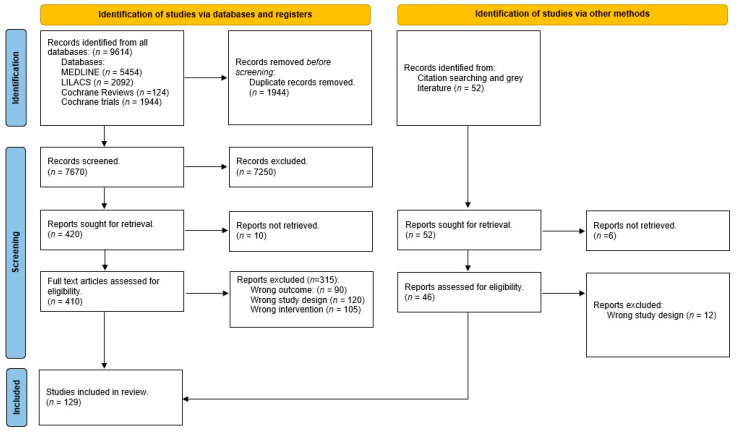
PRISMA flow diagram of the scoping review inclusion process.

We initially identified a total of 9614 publications from all the selected databases (Medline, LILACS, and Cochrane Library). Additionally, we found 52 articles through citation searches and grey literature. After removing duplicates (*n* = 1944), a total of 7670 publications from the different search strategies were manually screened. Based on titles and abstracts, we excluded 7250 publications, resulting in 420 articles. Of these, 10 could not be retrieved, leaving 410 full-text articles to be extracted and assessed for eligibility. From this group, we excluded 315 studies for the reasons outlined in Figure 1.

In addition to the database search, we identified 52 publications from citation searching, 6 of which were not retrieved. Of the 46 studies assessed for eligibility, 12 were excluded because they did not meet the eligibility criteria in relation to the type of study, in this case, opinion articles.

As a result, 129 studies were included in the review. The study inclusion process is shown in Figure 1.

### 3.2. Description of the Included Studies

The main characteristics of the studies included are presented in Supplementary Table S1.

**Figure 2:**
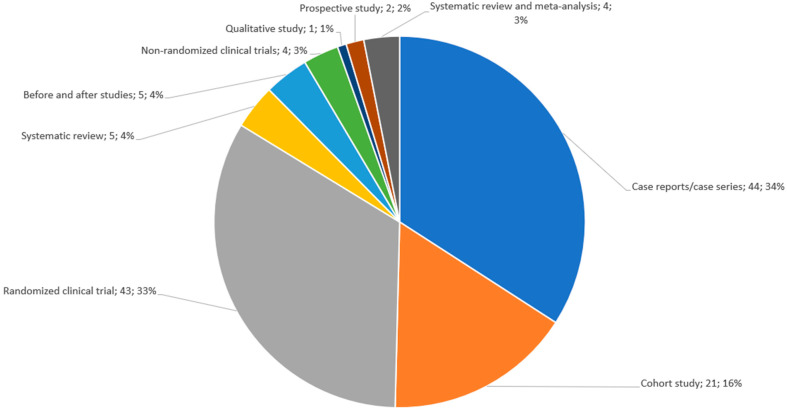
Distribution of included papers based on study type.

Regarding the types of included studies, there were 49 (36%) clinical trials, both randomized and non-randomized, 71 (57%) observational studies, and 9 (7%) systematic reviews. Among the seventy-one observational studies included, 44 (34%) were case reports/case series, 21 (16%) were cohort studies, 1 (1%) was a qualitative study, 2 (2%) were prospective observational studies, and 5 (4%) were before-and-after studies. Figure 2 shows the distribution of studies by type. The median number of patients was 44 (17, 78.5), with the highest numbers corresponding to the population within the systematic reviews. However, patient population was not available for all the selected studies. All publications had a therapeutic purpose, and adverse effects related to the use of LAs and the assessment of their allergenicity were also found. The most commonly reported LAs were lidocaine (*n* = 54), procaine (*n* = 36), and bupivacaine (*n* = 30).

Most of the studies were conducted in Türkiye (*n* = 24), United States (*n* = 19), Germany (*n* = 11), Cuba (*n* = 10), Spain (*n* = 10), Switzerland (*n* = 7), Korea (*n* = 6), and China (*n* = 8). The remaining publications hailed from 18 other countries, including Canada, Scotland, Japan, Italy, New Zealand, Colombia, Brazil, Venezuela, Greece, Hungary, Denmark, United Kingdom, Belgium, Russia, Lebanon, and India.

The median publication year was 2016, with an interquartile range from 2010 to 2019, indicating that over half of the selected studies were conducted between 2011 and March 2022.

In total, 63.5% percent of the articles were published in general medical journals. Specialty journals were identified, such as pain management journals (17.05%, *n* = 22), neurology or psychiatry journals (10.85%, *n* = 14), and anesthesiology journals (8.52%, *n* = 11), among others.

### 3.3. Description of Indications

**Figure 3:**
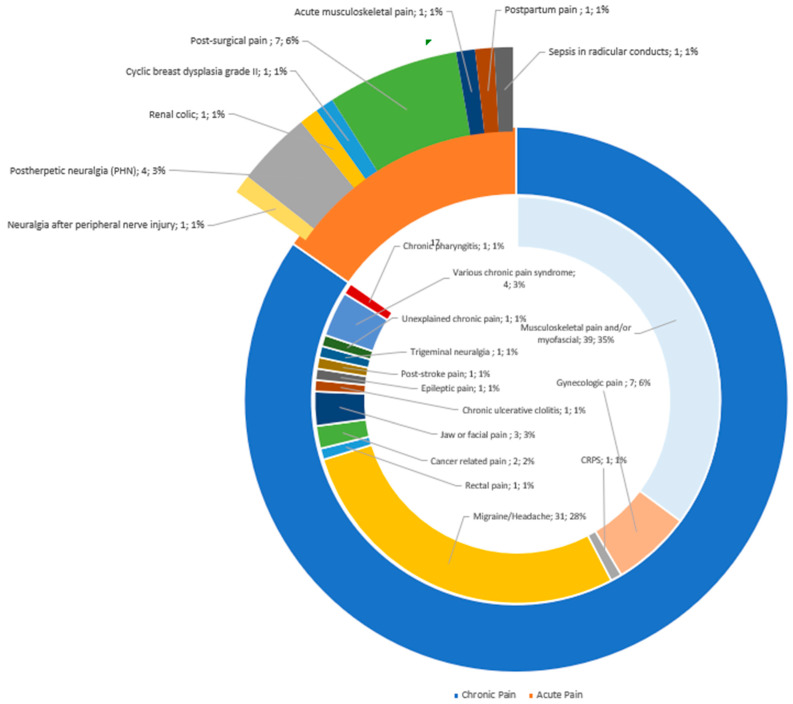
Therapy indications and main topics of included papers. CRPS: Complex regional pain syndrome.

The primary indication for low-dose, short-acting LAs was chronic pain (73%, *n* = 94). Additionally, acute pain was identified (13%, *n* = 17). Within the category of chronic pain, the most prevalent conditions were musculoskeletal and/or myofascial pain (*n* = 39), followed by migraine/headache (*n* = 31). Figure 3 illustrates the various therapeutic indications for both chronic and acute pain.

**Figure 4:**
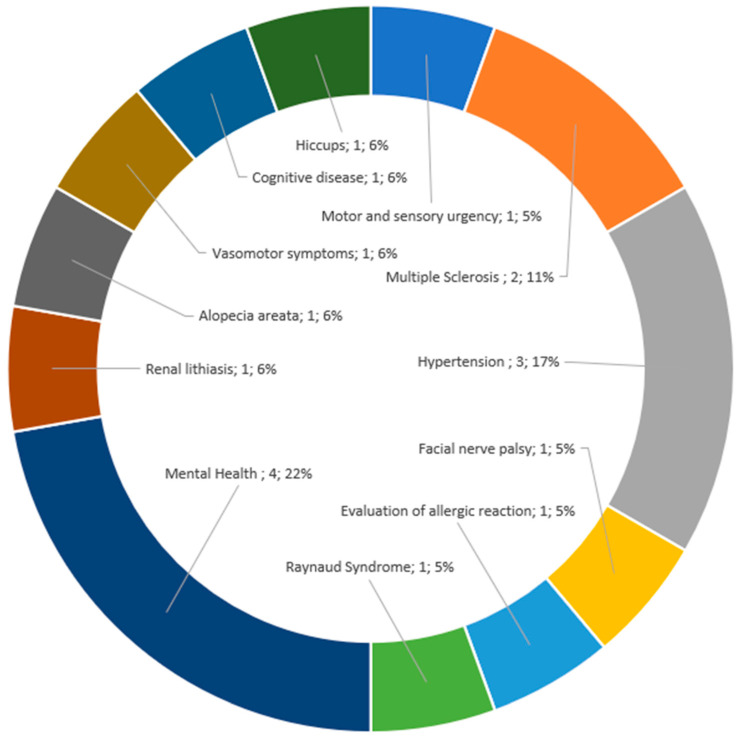
Non-pain indications.

We also found low-dose LAs were used for non-pain indications (14%, *n* = 18). Figure 4 shows the various non-pain indications identified.

In the collected studies, 47.28% (*n* = 61) reported the use of low doses of LAs for segmental application based on viscerocutaneous reflexes and for ganglion injections. Meanwhile, approximately 18.60% (*n* = 24) reported its use for local application or “loco dolendi”, or with trigger point infiltration, and 21.70% (*n* = 28) performed ganglion application looking for systemic effects. Overall, 10.35% (*n* = 14) of studies documented a mixed application of LAs, combining local, segmental therapy and interference field (neuromodulatory trigger^18^) treatment.

**Figure 5:**
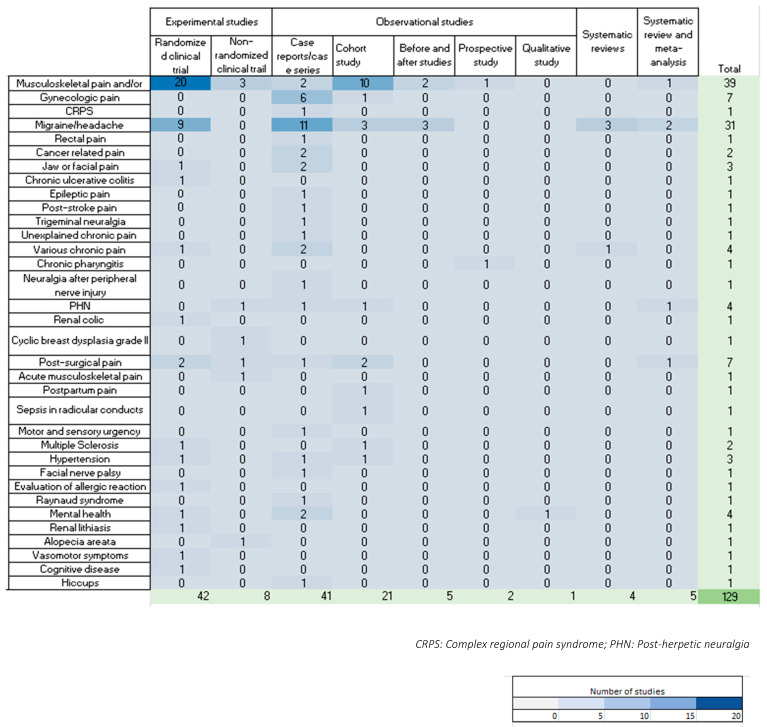
The current evidence gaps identified in the literature focusing on type of indication and type of study. CRPS: Complex regional pain syndrome; PHN: Post-herpetic neuralgia.

Figure 5 presents the current evidence gaps identified on the literature focusing on type of indication and type of study.

### 3.4. Outcomes

Our investigation encompassed a total of 124 studies (96.12%) that primarily assessed the efficacy of LAs, while five studies (3.87%) specifically focused on safety aspects. The most prevalent outcomes identified within the efficacy-focused studies included substantial statistics on pain relief (41.6%), improvements in patient quality of life (8.3%), reduced analgesic intake (6.25%), and improved scores on anxiety and depression scales (6.25%).

Within the cohort of studies evaluating efficacy, a notable subset—69 studies (53.48%)—considered safety as a secondary aspect. Of these, 18 studies (26.08%) reported transient, mild adverse reactions. These included localized symptoms, such as pain and itching at the injection site, and self-limiting vasomotor symptoms, such as nausea, dizziness, and numbness. It is pivotal to highlight that no severe adverse reactions or allergic responses were documented in any of the studies reviewed. Procaine and lidocaine emerged as the most extensively researched local anesthetics within the gathered studies. In terms of safety and the occurrence of adverse effects, studies involving lidocaine exhibited a higher frequency of reported events compared to those involving procaine, although neither showed any major adverse effects.

**Figure 6:**
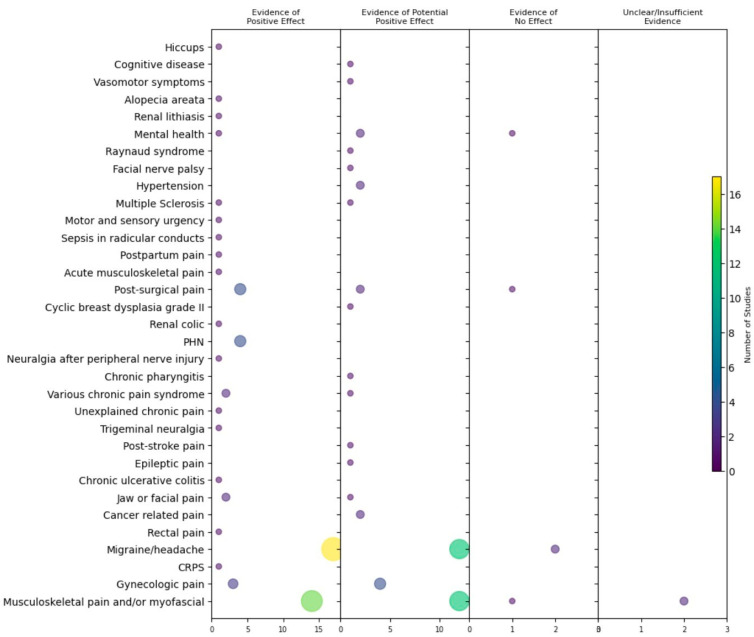
Evidence map by the intervention effect. The color and size of the bubble refer to the number of studies identified by indication and the effect of the intervention. CRPS: Complex regional pain syndrome; PHN: Post-herpetic neuralgia.

LA injections for therapeutic purposes were frequently reported to have a positive (48.06%) or potentially positive effect (36.43%). Only a small percentage of studies reported no effect or provided unclear/insufficient evidence (8.52% and 3.87%, respectively). Figure 6 shows the intervention effect by therapeutic indication and identifies evidence gaps in the current literature.

Regarding studies exploring the impact of LAs on acute stress, positive effects were reported. Regarding stress, anxiety, or depression as secondary outcomes (*n* = 4), one study demonstrated an improvement in anxiety or depression, two suggested a potential positive effect, while one reported no significant improvement in anxiety or depression.

## 4. Discussion

Our review was designed to consolidate the existing scientific knowledge on the therapeutic use of low-dose LAs for various conditions and to identify areas that warrant further investigation. Historically, the scope and design of studies evaluating the therapeutic efficacy and safety of LAs have remained largely unknown. This scoping review highlights emerging evidence that underscores the versatility of LAs beyond their traditional anesthetic role.

Our findings provide strong support for the therapeutic use of LAs in a variety of conditions. While pain, anxiety, and depression emerged as predominant concerns, LAs also demonstrated efficacy in the treatment of neuralgia, various chronic pain syndromes, renal colic, lithiasis, and more. Their role in alleviating stress, both acute and post-traumatic, is consistent with the principles of neural therapy, a neuroregulation therapy, which emphasizes the regulatory function of the ANS. By targeting specific neural regions with LAs, a regulatory process is generated that can help improve physical, mental, and emotional states.

### 4.1. Properties of LAs and Mechanisms of Action

LAs work by blocking voltage-gated sodium channels, inhibiting nerve conduction and thereby producing local analgesia. Originally developed for surgical applications, newer LAs have been synthesized to prolong the duration of anesthesia. However, therapeutic regulation does not depend on the prolonged action of LAs. Rather, it is the ability to “reset” pathological conduction that matters, regardless of how long this interruption lasts [17,21]. LAs are an important therapeutic option in the analgesic management of pain. Their use in pain relief strategies is logical because they effectively block the conduction of peripheral nociceptive nerve fibers, thereby attenuating the activity of the entire nociceptive system. This includes dampening the response of wide dynamic range (WDR) neurons in the spinal cord and attenuating central sensitization [16,54].

Over time, LAs have been found to exert a range of alternative effects, including modulation of the inflammatory cascade, inhibition of inflammatory mediators, antiarrhythmic [55], protection against thromboembolism [56], antimicrobial activity, and even anticancer properties [57,58,59,60,61,62].

LAs exhibit powerful anti-inflammatory effects acting on both humoral and cellular levels [62], potentially surpassing traditional steroidal or non-steroidal anti-inflammatory agents in efficacy and tolerability [63]. They reduce inflammation while preserving the body’s healing and protective responses, effectively managing pain without delaying healing or increasing the risk of infection [64]. Furthermore, lower concentrations of LAs can maintain an anti-inflammatory and, consequently, analgesic effect, minimizing the risk of potential toxicity [65].

Beyond the immediate effects of LAs and their alternative properties, it is necessary to consider other mechanisms to account for the sustained therapeutic outcomes observed in conditions like therapy-resistant pain and inflammation. The role of the ANS is important in this context. Our previous research postulates the existence of positive feedback loops in pain and inflammatory pathways controlled by the ANS [17,18,25,65]. Interestingly, these basic feedback mechanisms are universal and can be activated by various triggers such as infection, mechanical trauma, or psychological stress [13,17,28,66]. Consequently, the results of our study elucidate why a single agent, such as LAs, can be therapeutically effective in a wide range of clinical diagnoses. In the following sections, we show the importance of the ANS in the emergence of feedback loops and their interruption by LAs.

#### 4.1.1. Neuronal Reflex Circuitries

The anatomical organization of neuronal pathways, characterized by principles of convergence and divergence, exemplifies a fundamental biological mechanism that facilitates positive feedback loops within reflex circuits [17]. A prime example of this is the convergence of visceral and somatic nociceptive afferents on the same multireceptive neurons in the dorsal horn of the spinal cord (wide dynamic range WDR neurons). Subsequent fibers give rise to divergent efferent pathways that project to: (1) higher brain centers, (2) sympathetic or parasympathetic nuclei within the lateral horn, (3) the anterior horn, innervating the skeletal musculature [5]. Clinically, this can result in increased peripheral myofascial tone and skin turgor, changes in microcirculation, and hyperalgesia, indicating sensitization processes in which the sympathetic nervous system plays a central role. The resulting peripheral sensitization enhances sympathetic hyperactivity, thereby amplifying nociceptive input to the spinal cord [65]. This reciprocal reinforcement between peripheral and central mechanisms constitutes a positive feedback loop involving the ANS [25]. Interventionally, the transient disruption of these reflexive pathways through the administration of LAs may facilitate a reversion to physiological homeostasis [17,20,25]. The strategic application of LAs can be tailored to the individual by targeting specific anatomical structures such as trigger points, scars, sympathetic ganglia, and peripheral nerves. The repeated blockade of sensitized nociceptive afferents by LAs contributes to the modulation of neuroplastic changes within neuronal centers, potentially attenuating “pain memory” [65]. In addition, useful combinations of injections can be applied simultaneously, having a positive effect on inhibitory mechanisms, such as the gate control of pain postulated by Melzack and Wall [67], reducing nociceptive transmission at the dorsal horn.

#### 4.1.2. Pathophysiological Coupling Mechanisms between Sympathetic and Nociceptive Systems

Under pathological conditions, a short-circuit may occur between sympathetic efferent fibers and nociceptive afferents, a phenomenon referred to as “sympathetic afferent coupling” [16,68]. Nociceptive afferents [68,69,70], and even immune cells [71], can express adrenergic receptors. This expression facilitates a pathological nexus where the efferent sympathetic outflow gains the ability to directly activate the afferent nociceptive pathways as well as modulate immune responses, creating a cascade that can potentiate pain and inflammation, thereby establishing a self-perpetuating positive feedback loop [17].

A similar process is called “sympathetic sprouting,” wherein sympathetic fibers undergo morphological changes, elaborating basket-like structures in the dorsal root ganglia of nociceptive afferents under inflammatory and neuropathic conditions [72,73,74]. Such structural reorganization allows for an enhanced and rapid nociceptive response to sympathetic stimuli (peripheral or central), thereby exacerbating pain in a positive feedback loop [5]. LAs have been shown to reduce sympathetic sprouting in dorsal root ganglia with increased spontaneous activity [75,76].

#### 4.1.3. Sensitization and Neuroplastic Mechanisms

Sensitization processes can induce peripheral and central neuroplastic changes [77], effectively allowing the nervous system to acquire and retain new functional states like learning and memory. This capacity for neuroadaptation extends to the sympathetic ganglia, where it can result in the enhancement of the postsynaptic neuronal response to repetitive presynaptic activity (synaptic long-term potentiation LTP) [78,79,80], showing the efficacy of LAs in indirectly reducing LTP.

Thus, the sympathetic nervous system can engrammatically store “old” stimuli and respond to new physiological stimuli with an overshooting pathological response [17,65,66,78,81,82]. This can be seen as a memory and learning process [28,77,82]. Any activation of the sympathetic system, peripheral or central (including emotions), can amplify symptomatic manifestations, such as pain or inflammation, by establishing positive feedback loops [17,24]. Repeated LA-induced blockade of sensitized nociceptive afferent neurons also allows modulation of plastic changes in neuronal centers (“pain memory”) [65].

#### 4.1.4. Neuroimmunological Interactions and Therapeutic Implications

The ANS and the immune system are in a dynamic and intricate communication network, as evidenced by extensive research [13,15,17,19,25,26,27,28,83]. These reflectory neuroimmunological and inflammatory cascades constitute a general reaction principle of the organism under the leadership of the ANS [17]. We could detect several interdependent positive feedback loops that can exacerbate inflammation and pain [17].

Crucially, these feedback loops depend strongly on the ANS, with sympathetic efferents playing an important role alongside the modulatory influence of cytokines and vagal fibers interacting within the sympathetic centers of the brainstem [17]. These loops operate simultaneously, reinforcing each other and perpetuating the physiological response [17].

A remarkable aspect of these neuroimmune interactions is their consistency across disease states, regardless of the nature of the initial stimulus—be it infection, mechanical trauma, or psychological stress [13,16,17,28,66]. This consistency suggests that basic pathological neuroimmune communication can be effectively modulated by LAs, particularly through interventions such as stellate ganglion blocks, which can “reset” these dysregulated pathways [17].

Consequently, we postulated a unified pathogenetic mechanism within the neuroimmune system, predominantly under the regulation of the sympathetic nervous system [17]. This common principle explains the efficacy of stellate ganglion blocks with LA in the treatment of a wide range of conditions such as acute and chronic pain, acute respiratory distress syndrome, pneumonia, traumatic brain injury, post-traumatic stress disorder, autoimmune diseases, heart failure including arrhythmias, microcirculatory disorders, autonomic dysfunction, neurogenic inflammation, complex regional pain syndrome, early systemic inflammatory response in severe trauma, and more [17,19,38,39,84,85,86,87,88,89,90,91].

### 4.2. Safety

No significant adverse effects from LAs toxicity were found in the reviewed articles, which is consistent with the established literature that consistently emphasizes the dose-dependent nature of toxicity. The administration of LAs at low doses is remarkably safe. While there have been historical concerns about potential allergic reactions to LAs, particularly procaine, our review and recent research have largely allayed these fears. In our review, lidocaine and procaine emerged as the predominant LAs of choice, with their selection likely influenced by their relatively low toxicity profiles. The prevalence of lidocaine may be attributed to its widespread availability and lower incidence of allergic reactions. However, when evaluated purely from a toxicity standpoint, procaine may be the more prudent choice due to its even lower toxicity, shorter half-life, and lack of reliance on hepatic metabolism, particularly in therapeutic contexts without anesthetic intent.

Any needle-based procedure carries inherent risks associated with puncture, including potential complications such as severe hematoma and pneumothorax. LAs in the cerebrospinal fluid space can compromise vital functions. Importantly, the severity of these complications is dose-related. Nevertheless, our study shows a high level of safety, as no complications were reported in the studies reviewed.

This safety, combined with their proven efficacy, underscores the importance of exploring LAs for novel therapeutic applications.

### 4.3. Limitations of Our Scoping Review

Our study has some limitations. From our initial review of over 9000 publications, a significant majority had to be excluded. The wide range of methodological and design variations, particularly evident in publications spanning several decades, introduces considerable variability into the literature. Given that the therapeutic use of LAs spans over a century, this variability can be viewed as a characteristic feature of the literature rather than a strict limitation. Nonetheless, it is essential to acknowledge that this variability presents challenges in drawing definitive conclusions. While recognizing these inherent limitations, it is important to emphasize that our review is at the forefront of consolidating the literature on the therapeutic use of LAs within a unified methodological framework.

### 4.4. Future

Recent and historical research underscores the diverse therapeutic potential of LAs beyond their primary anesthetic role. Modern academic institutions are increasingly exploring the therapeutic use of low-dose LAs, particularly in areas such as pain management, inflammation, dysfunction, and other conditions. The strategy of drug repurposing offers a promising avenue for these agents, given their minimal side effects, negligible drug interactions, affordability, and global availability. The remarkable beneficial effects of LAs are increasingly being investigated in relation to additional comorbidities, such as cancer, as evidenced by Badwe et al. and other referenced studies [60]. While their safety and efficacy are well established, there is an urgent need for increased research into the expansive therapeutic applications of local anesthetics.

Our review highlights the need for high-quality observational studies to complement clinical trials and provide a broader, comprehensive societal perspective.

## 5. Conclusions

In summary, our systematic scoping review highlights the versatile therapeutic potential of LAs in a variety of clinical contexts and known mechanisms of action. When used judiciously, LAs have consistently demonstrated safety and efficacy in the management of pain, inflammation, and other conditions and related symptoms, with no significant adverse effects. More rigorous studies are needed to further elucidate pathophysiologic mechanisms and to identify new therapeutic avenues. Future research should prioritize determining optimal dosing, refining treatment methodologies, and evaluating long-term outcomes.

## Data Availability

Data supporting the reported results can be found using the public PUBMED/Medline, Cochrane Library and LILACS databases.

## Supplementary Materials

The following supporting information can be downloaded at: https://www.mdpi.com/article/10.3390/jcm12237221/s1, Annex A: Preferred Reporting Items for Systematic reviews and Meta-Analyses extension for Scoping Reviews (PRISMA-ScR) Checklist; Annex B: Terms used to defined the search strategy; Table S1: The main characteristics of the studies included.

## References

[1] Koller C. (1928). Personal reminiscences of the first use of cocaine as local anesthetic in eye surgery. Curr. Res. Anesth. Analg..

[2] Spiess G. (1906). Die Bedeutung der Anästhesie in der Entzündungstheorie. Münch Med. Wschr..

[3] Weinschenk S. (2012). Neural therapy-Areview of the therapeutic use of local anesthetics. Acupunct. Relat. Ther..

[4] Barop H. (2018). Textbook and Atlas of Neural Therapy: Diagnosis and Therapy with Local Anesthetics.

[5] Fischer L. (2019). Neuraltherapie: Neurophysiologie, Injektionstechnik und Therapievorschläge.

[6] Huneke F. (1928). Unbekannte Fernwirkung der Neuraltherapie. Die Med. Welt.

[7] Leriche R., Fontaine R. (1934). L’anesthésie du ganglion étoile: Sa technique, ses indications, ses résultats. Presse Méd..

[8] Dosch P. (1995). Lehrbuch der Neuraltherapie nach Huneke.

[9] Fischer L., Barop H., Maxion-Bergemann S. (2005). Health Technology Assessment HTA Neural Therapy.

[10] Mermod J., Fischer L., Staub L., Busato A. (2008). Patient satisfaction of primary care for musculoskeletal diseases: A comparison between Neural Therapy and conventional medicine. BMC Complement. Altern. Med..

[11] Dönges A., Fischer L., Marian F., Widmer M., Herren S., Busato A. (2005). Evaluation of Neural Therapy and Comparison with Conventional Medicine: Structure, Process an Outcomes. A Nationwide Evaluation Funded by the Swiss Federal Office of Public Health. Ph.D. Thesis.

[12] Bissig P., Schoeni-Affolter F., Fischer L., Busato A. (2008). Is Neural Therapy Cheaper than Conventional Medicine? A Comparison of Cost Structure in Swiss Primary Care Providers. An Observational Study as a Part of a Nationwide Evaluation Funded by the Swiss Federal Office of Public Health. Ph.D. Thesis.

[13] Elenkov I.J., Wilder R.L., Chrousos G.P., Vizi E.S. (2000). The sympathetic nerve–an integrative interface between two supersystems: The brain and the immune system. Pharmacol. Rev..

[14] Tracey K.J. (2009). Reflex control of immunity. Nat. Rev. Immunol..

[15] Pavlov V.A., Chavan S.S., Tracey K.J. (2018). Molecular and Functional Neuroscience in Immunity. Annu. Rev. Immunol..

[16] Jänig W. (2022). The Integrative Action of the Autonomic Nervous System.

[17] Fischer L., Barop H., Ludin S.M., Schaible H.G. (2022). Regulation of acute reflectory hyperinflammation in viral and other diseases by means of stellate ganglion block. A conceptual view with a focus on COVID-19. Auton. Neurosci..

[18] Engel R., Barop H., Giebel J., Ludin S.M., Fischer L. (2022). The Influence of Modern Neurophysiology on the Previous Definitions of “Segment” and “Interference Field” in Neural Therapy. Complement. Med. Res..

[19] Fleckenstein J., Neuberger E.W.I., Bormuth P., Comes F., Schneider A., Banzer W., Fischer L., Simon P. (2021). Investigation of the Sympathetic Regulation in Delayed Onset Muscle Soreness: Results of an RCT. Front. Physiol..

[20] Puente de la Vega K., Gómez M., Roqueta C., Fischer L. (2016). Effects on hemodynamic variables and echocardiographic parameters after stellate ganglion block in 15 healthy volunteers. Auton. Neurosci..

[21] Egli S., Pfister M., Ludin S.M., Puente de la Vega K., Busato A., Fischer L. (2015). Long-term results of therapeutic local anesthesia (neural therapy) in 280 referred refractory chronic pain patients. BMC Complement. Altern. Med..

[22] Kronenberg R.M., Ludin S.M., Fischer L. (2018). Severe case of chronic pelvic pain syndrome: Recovery after injection of procaine into the vesicoprostatic plexus-case report and discussion of pathophysiology and mechanisms of action. Case Rep. Urol..

[23] Shiratori Tusita L.N., Fischer L. (2023). Chronic Therapy-Resistant Neck Pain in a Fifty-Year-Old Man: The Role of Partially Impacted Third Molars—Case Report and New Pathophysiological Insights. Complement. Med. Res..

[24] Lopes C.A., Fischer L. (2023). A case of severe trigeminal neuralgia: Recovery by means of stellate ganglion block with procaine. Discussion of possible mechanisms of action. J. Int. Med. Res..

[25] Fischer L. (2003). Pathophysiology of pain and neural therapy. Praxis.

[26] Schaible H.G. (2014). Nociceptive neurons detect cytokines in arthritis. Arthritis Res. Ther..

[27] Schaible H.G., Straub R.H. (2014). Function of the sympathetic supply in acute and chronic experimental joint inflammation. Auton. Neurosci..

[28] Tracey K.J. (2002). The inflammatory reflex. Nature.

[29] Strong J.A., Zhang J.M., Schaible H.G., Wood J.N. (2018). The sympathetic nervous system and pain. The Oxford Handbook of the Neurobiology of Pain.

[30] Vinyes D., Muñoz-Sellart M., Caballero T.G. (2022). Local anesthetics as a therapeutic tool for post COVID-19 patients: A case report. Medicine.

[31] Rey M., Muñoz M., Catalán M., Vinyes D. (2021). Treatment of Localized Vulvar Pain with Neural Therapy: A Case Series and Literature Review. Complement. Med. Res..

[32] Bashan I., Ozturk G.Y. (2022). Effect of Neural Therapy on shoulder dysfunction and pain in supraspinatus tendinopathy. Pak. J. Med. Sci..

[33] Nazlıkul H., Ural F.G., Öztürk G.T., Öztürk A.D.T. (2018). Evaluation of neural therapy effect in patients with piriformis syndrome. J. Back Musculoskelet. Rehabil..

[34] Feigin G., Velasco Figueroa S., Englesakis M.F., D’Souza R., Hoydonckx Y., Bhatia A. (2023). Stellate Ganglion Block for Non-Pain Indications: A Scoping Review. Pain Med..

[35] Fischer L., Ludin S.M., Thommen D., Hausammann R. (2010). Application for the Adoption of Interference Field Therapy (Huneke’s Neural Therapy) into the Health Benefit Basket of the Compulsory Health Insurance, submitted to the Federal Commission for General Health Insurance Benefits and Fundamental Health Issues and the Federal Department of Home Affaires (EDI) in the Federal Office of Public Health (BAG).

[36] Barbagli P., Bollettin R. (1998). Therapy of articular and periarticular pain with local anesthetics (neural therapy a. t. Huneke). Long and short term results. Minerva Anesth..

[37] Gibson R.G., Gibson S.L. (1999). Neural therapy in the treatment of multiple sclerosis. J. Altern. Complement. Med..

[38] Liu M.H., Tian J., Su Y.P., Wang T., Xiang Q., Wen L. (2013). Cervical sympathetic block regulates early systemic inflammatory response in severe trauma patients. Med. Sci. Monit..

[39] Yang X., Shi Z., Li X., Li J. (2015). Impacts of stellate ganglion block on plasma NF-κB and inflammatory factors of TBI patients. Int. J. Clin. Exp. Med..

[40] Zhao H.Y., Yang G.T., Sun N.N., Kong Y., Liu Y.F. (2017). Efficacy and safety of stellate ganglion block in chronic ulcerative colitis. World J. Gastroenterol..

[41] Rahimzadeh P., Mahmoudi K., Khodaverdi M. (2020). Faiz SHR: Effects of ultrasound guided ganglion stellate blockade on intraoperative and postoperative hemodynamic responses in laparoscopic gynecologic surgery. Videosurgery Miniinv.

[42] Triantafyllidi H., Arvaniti C., Schoinas A., Benas D., Vlachos S., Palaiodimos L., Pavlidis G., Ikonomidis I., Batistaki C., Voumvourakis C. (2018). Bilateral sphenopalatine ganglion block reduces blood pressure in never treated patients with essential hypertension. A randomized controlled single-blinded study. Int. J. Cardiol..

[43] Kahokehr A., Sammour T., Soop M., Hill A.G. (2011). Intraperitoneal local anaesthetic in abdominal surgery—A systematic review. ANZ J. Surg..

[44] Atalay N.S., Sahin F., Atalay A., Akkaya N. (2013). Comparison of efficacy of neural therapy and physical therapy in chronic low back pain. Afr. J. Tradit. Complement. Altern. Med..

[45] Lynch J.H., Mulvaney S.W., Kim E.H., de Leeuw J.B., Schroeder M.J., Kane S.F. (2016). Effect of Stellate Ganglion Block on Specific Symptom Clusters for Treatment of Post-Traumatic Stress Disorder. Mil. Med..

[46] Naja Z., Al-Tannir M., El-Rajab M., Ziade F., Baraka A. (2009). Nerve stimulator-guided occipital nerve blockade for postdural puncture headache. Pain Pract..

[47] Tepe N., Tertemiz O.F. (2021). Comparison of greater occipital nerve and greater occipital nerve + supraorbital nerve block effect in chronic medication overuse headache. Turk. J. Med. Sci..

[48] Kim H.J., Ahn H.S., Lee J.Y., Choi S.S., Cheong Y.S., Kwon K., Yoon S.H., Leem J.G. (2017). Effects of applying nerve blocks to prevent postherpetic neuralgia in patients with acute herpes zoster: A systematic review and meta-analysis. Korean J. Pain.

[49] Zhang H., Yang X., Lin Y., Chen L., Ye H. (2018). The efficacy of greater occipital nerve block for the treatment of migraine: A systematic review and meta-analysis. Clin. Neurol. Neurosurg..

[50] Mellick L.B., Mellick G.A. (2008). Treatment of acute orofacial pain with lower cervical intramuscular bupivacaine injections: A 1-year retrospective review of 114 patients. J. Orofac. Pain.

[51] Vinyes D., Hermosilla Traverso P., Murillo J.H., Sánchez-Padilla M., Muñoz-Sellart M. (2023). Improvement in post-orthodontic chronic musculoskeletal pain after local anesthetic injections in the trigeminal area: A case series. J. Int. Med. Res..

[52] Tricco A.C., Lillie E., Zarin W., O’Brien K.K., Colquhoun H., Levac D., Moher D., Peters M.D.J., Horsley T., Weeks L. (2018). PRISMA Extension for Scoping Reviews (PRISMA-ScR): Checklist and Explanation. Ann. Intern. Med..

[53] Huang X., Lin J., Demner-Fushman D. (2006). Evaluation of PICO as a knowledge representation for clinical questions. AMIA Annu. Symp. Proc..

[54] Jänig W., Baron R., Fischer L., Peuker E.T. (2011). Pathophysiologie des Schmeruzes. Lehrbuch der Integrativen Schmerztherapie.

[55] Borgeat A., Aguirre J. (2010). Update on local anesthetics. Curr. Opin. Anaesthesiol..

[56] Modig J. (1989). Influence of regional anesthesia, local anesthetics, and sympathicomimetics on the pathophysiology of deep vein thrombosis. Acta Chir. Scand. Suppl..

[57] Villar-Garea A., Fraga M.F., Espada J., Esteller M. (2003). Procaine is a DNA-demethylating agent with growth-inhibitory effects in human cancer cells. Cancer Res..

[58] Gradinaru D., Ungurianu Am Margina D., Villanueva M.M., Bürkle A. (2021). Procaine-The Controversial Geroprotector Candidate: New Insights Regarding its Molecular and Cellular Effects. Oxid. Med. Cell. Longev..

[59] Zhu G., Zhang L., Dan J., Zhu Q. (2020). Differential effects and mechanisms of local anesthetics on esophaeal carcinoma cell migration, growth, survival and chemosensitivity. BMC Anesthesiol..

[60] Badwe R.A., Parmar V., Nair N., Joshi S., Hawaldar R., Pawar S., Kadayaprath G., Borthakur B.B., Thammineedi S.R., Pandya S. (2023). Effect of Peritumoral Infiltration of Local Anesthetic Before Surgery on Survival in Early Breast Cancer. J. Clin. Oncol..

[61] Hollmann M.W., Durieux M.E. (2000). Local anaesthetics and the inflammatory response: A new therapeutic indication?. Anaesthesiology.

[62] Cassuto J., Sinclair R., Bonderovic M. (2006). Anti-inflammatory properties of local anesthetics and their present and potential clinical implications. Acta Anaesthesiol. Scand..

[63] Beilin B., Shavit Y., Trabekin E., Mordashev B., Mayburd E., Zeidel A., Bessler H. (2003). The effects of postoperative pain management on immune response to surgery. Anesth. Analg..

[64] Lirk P., Picardi S., Hollmann M.W. (2014). Local anaesthetics: 10 essentials. Eur. J. Anaesthesiol..

[65] Pietruck C., Grond S., Xie G.X., Palmer P.P. (2003). Local anesthetics differentially inhibit sympathetic neuron-mediated and C fiber-mediated synovial neurogenic plasma extravasation. Anesth. Analg..

[66] Ricker G. (1924). Pathologie als Naturwissenschaft, Relationspathologie.

[67] Melzack R., Wall P.D. (1965). Pain mechanisms; a new theory. Science.

[68] Baron R., Jänig W. (1998). Schmerzsyndrome mit kausaler Beteiligung des Sympathikus. Anaesthesist.

[69] Sato J., Perl E.R. (1991). Adrenergic excitation of cutaneous pain receptors incuced by peripheral nerve injury. Science.

[70] Birder L.A., Perl E.R. (1999). Expression of alpha2-adrenergic receptors in rat primary afferent neurones after peripheral nerve injury or inflammation. J. Physiol..

[71] Bellinger D.L., Lorton D. (2014). Autonomic regulation of cellular immune function. Auton. Neurosci..

[72] McLachlan E.M., Jänig W., Devor M., Michaelis M. (1993). Peripheral nerve injury triggers noradrenergic sprouting within dorsal root ganglia. Nature.

[73] Chung K., Chung J.M. (2001). Sympathetic sprouting in the dorsal root ganglion after spinal nerve ligation: Evidence of regenerative collateral sprouting. Brain Res..

[74] Ramer M.S., Bisby M.A. (1997). Rapid sprouting of sympathetic axons in dorsal root ganglia of rats with a chronic constriction injury. Pain.

[75] Takatori M., Kuroda Y., Hirose M. (2006). Local anesthetics suppress nerve growth factor-mediated neurite outgrowth by inhibition of tyrosine kinase activity of TrkA. Anesth. Analg..

[76] Zhang J.M., Li H., Munir M.A. (2004). Decreasing sympathetic sprouting in pathologic sensory ganglia: A new mechanism for treating neuropathic pain using lidocaine. Pain.

[77] Sandkühler J. (2000). Learning and memory in pain pathways. Pain.

[78] Alkadhi K.A., Alzoubi K.H., Aleisa A.M. (2005). Plasticity of synaptic transmission in autonomic ganglia. Prog. Neurobiol..

[79] Kansha M., Nagata T., Irita K., Takahashi S. (1999). Dibucaine and tetracaine inhibit the activation of mitogen-activated protein kinase mediated by L-type calcium channels in PC12 cells. Anesthesiology.

[80] Tan Z., Dohi S., Ohguchi K., Nakashima S., Nozawa Y. (1999). Local anaesthetics inhibit muscarinic receptor-mediated activation of extracellular sign regulated kinases in rat feochromocytoma PC12 cells. Anesthesiology.

[81] Eggli P., Fischer L., Fischer Land Peuker E.T. (2011). Vegetatives Nervensystem (Neuroanatomische und neurophysiologische Grundlagen). Lehrbuch Integrative Schmerztherapie.

[82] McLachlan E. (2003). Transmission of signals through sympathetic ganglia—Modulation, integration or simply distribution?. Acta Physiol. Scand..

[83] Schaible H.G., Ebersberger A., Natura G. (2011). Update on peripheral mechanisms of pain: Beyond prostaglandins and cytokines. Arthritis Res. Ther..

[84] Kumar N., Thapa D., Gombar S., Ahuja V., Gupta R. (2014). Analgesic efficacy of pre-operative stellate ganglion block on postoperative pain relief: A randomised controlled trial. Anaesthesia.

[85] García-Morán E., Sandín-Fuentes M.G., Álvarez López J.C., Duro-Aguado I., Urueña-Martínez N., Hernández-Luis C. (2013). Electrical storm secondary to acute myocardial infarction and heart failure treated with left stellate ganglion block. Rev. Esp. Cardiol..

[86] Guo W., Jin X.J., Yu J., Liu Y., Zhang J.P., Yang D.W., Zhang L., Guo J.-R. (2014). Effects of stellate ganglion block on the peri-operative vasomotor cytokine content and intrapulmonary shunt in patients with esophagus cancer. Asian Pac. J. Cancer Prev..

[87] Zhang L., Yao J., Zhang T., Jin J., Zeng X., Yue Z. (2013). Stellate ganglion block may prevent the development of neurogenic pulmonary edema and improve the outcome. Med. Hypotheses.

[88] Fischer L. (1995). Neuraltherapie in der Notfallmedizin. Ärztez f NHV.

[89] Patel R.A., Priore D.L., Szeto W.Y., Slevin K.A. (2011). Left Stellate Ganglion Blockade for the Management of Drug-Resistant Electrical Storm. Pain Med..

[90] Alino J., Kosatka D., McLean B., Hirsch K. (2013). Efficacy of stellate ganglion block in the treatment of anxiety symptoms from combat-related post-traumatic stress disorder: A case series. Mil. Med..

[91] Kirkpatrick K., Khan M.H., Deng Y., Shah K.B. (2023). A review of Stellate Ganglion Block as an Adjunctive Treatment Modality. Cureus.

