# Supplementary material for: Therapeutic Use of Low-Dose Local Anesthetics in Pain, Inflammation, and Other Clinical Conditions: A Systematic Scoping Review
Source: J Clin Med. 2023 Nov 21;12(23):7221. doi: 10.3390/jcm12237221 (PMC10707454; doi:10.3390/jcm12237221)
Supplement: Supplementary file 1 [file jcm-12-07221-s001.zip › jcm-2675459-supplementary/Annex B. Terms used to define the search strategy.pdf]

Annex B. Terms used to define the search strategy.

1. Neural therapy
2. Segmental therapy
3. Complementary therapies
4. Local Anesthetics
5. Lidocaine
6. Anodyne
7. Anodine
8. Procaine
9. Ropivicaine
10. Bupivicaine
11. Mepivicaine
12. Small amount
13. Low concentration
14. Subcutaneous injection
15. Intramuscular injection
16. Intradermal injection
17. Intravenous injection
18. Via nasal
19. Endovenous
20. Trigger points
21. Paravertebral
22. Infiltration
23. Interference field
24. Nerve block
25. Ganglion block
26. Stellate ganglion block
27. Sphenopalatine ganglion block
28. Celiac ganglion block
29. Hypogastric ganglion block
30. Pelvic ganglion block
31. Gasserian ganglion block
32. Nerve block
33. Cranial nerve block
34. Supraorbital nerve block
35. Infraorbital nerve block
36. Occipital nerve block
37. Mental nerve block
38. Mandibular nerve block
39. Maxillary nerve block
40. Trigeminal nerve block
41. Splanchnic nerve block
42. Ciliary block
43. Pudendal block
44. Ganglion Impar Block
45. Paravertebral sympathetic block
46. Cervical sympathetic block
47. Lumbar sympathetic block
