# Supplementary material for: Therapeutic Use of Low-Dose Local Anesthetics in Pain, Inflammation, and Other Clinical Conditions: A Systematic Scoping Review
Source: J Clin Med. 2023 Nov 21;12(23):7221. doi: 10.3390/jcm12237221 (PMC10707454; doi:10.3390/jcm12237221)
Supplement: Supplementary file 1 [file jcm-12-07221-s001.zip › jcm-2675459-supplementary/Table S1. The main characteristics of the studies included.pdf]

Vinyes D, Muñoz-Sellart M, Fischer L. Therapeutic Use of Low-Dose Local Anesthetics in Pain, Inflammation, and Other Clinical Conditions: A Systematic Scoping Review. *Journal of Clinical Medicine*. 2023; 12(23):7221. https://doi.org/10.3390/jcm12237221

Table S1. The main characteristics of the studies included.

					Type of intervention			
**Author**	**Year**	**Study Type**	**Population included**	**Indication**	**Intervention**	**Comparative group**	**Effect of the intervention**	**Type of evaluation**
Allen S. et al.	2018	Before and after study	562	Migraine	Buvacaine 0,25%, Bupivacaine 0,5%, Lidocaine 1%. Local infiltration	No comparative group	High rates of response to greater occipital nerve block, in decreased migraine pain intensity, are found, but randomized clinical trials are needed to confirm the findings.	Numeric Pain Rating Scale (NPRS). Minimal, moderate, or significant response groups according to decreased NPRS score 1 and 4 weeks after blockade
Affaitati G. et al.	2009	Randomized clinical trial	60	Myofascial pain syndrome	1 mL of 0.5% bupivacaine hydrochloride infiltration at trigger points	Lidocaine and placebo patches	Myofascial pain control with infiltration treatment but more comfort with patches treatment	Pain intensity scores
Altınbilek T. et al.	2019	Randomized clinical trial	60	Fibromyalgia	0.1-0.1 ml 0.55 of lidocaine Interspinous injection	Exercise programs	When NT is combined with exercise, improvement in pain and depression scores is documented	Pain assessed with VAS, emotional state with the Beck Depression Scale and Beck Anxiety Scale, and quality of life with the SF-36
Álvarez Urbay M. et al.	2007	Prospective study	71	Chronic pharyngitis	0.5 ml of 1% procaine submucosa infiltration	No comparative group	Satisfactory response to the treatment	Control of symptoms
Amhaz H. et al.	2013	Case report	1	Contralateral Horner´s syndrome	10 ml lidocaine 1,5% Intradermic injection	No comparative group	Considering neurovascular structures and uncontrolled migration of LA, surveillance should be performed for potential adverse events.	Description contralateral Horner Syndrome

Arnér S. et al.	1990	Case reports	38	Neuralgia after nerve injury	Peripheral anesthetic block with 5-10ml of 5% bupivacaine	No comparative group	No long-term effect	Neuralgia pain relief
Arthur B. et al.	2019	Case report	1	Dyspareunia in patients due to vaginal hysterectomy	4-5ml 0.5% of procaine infiltration	No comparative group	NT of vaginal hysterectomy scars may have a role in the treatment of dyspareunia.	Control of dyspareunia
Ashkenazi A. et al.	2010	Systematic review	157	Headache	Different concentrations and anesthetics and reports of combination steroid therapies	For the RCT comparative group: placebo	The results were generally positive but should be taken with caution given the methodological limitations of the available studies.	Migraine pain control with peripheral nerve block, adverse effects
Atalay NS. et al.	2013	Cohort study	60	Chronic low back pain	Lidocaine 20mg/ml	Physical therapy	This therapy is effective in pain relief, quality of life, disability, and depression	Pain relief scales, disability Questionnaire (RMDQ), quality of life with Nottingham-Health-Profile (NHP), depression assessed with HADS
Ay S. et al.	2010	Randomized clinical trial	80	Myofascial pain syndrome	2 ml 1% of lidocaine	Dry injection into trigger points	Potential positive effect	Pain, cervical movement, and depression assessment with scores/scales
Balevi Batur E. et al.	2021	Cohort study	60	Fibromyalgia	Lidocaine al 1%	Physical therapy	Neural therapy can be an effective treatment to improve quality of life in Fibromyalgia patients	Pain assessed with VAS, quality of life with SF 36 and Fibromyalgia Impact Questionnaire (FIQ)

Barad M. et al.	2022	Systematic review	14 studies	Migraine	Not applicable	Variable	1. Weak recommendation for major occipital nerve block for the management of chronic migraine compared with placebo, insufficient evidence. 2. Insufficient evidence for trigger point injection with LA compared with saline to reduce acute cephalea days	Generating recommendations
Boluk Senlikci H. et al.	2021	Case report	1	Temporomandibular disorders and dysfunction of sacro-iliac joint	1 ml 0.5% of lidocaine	No comparative group	The relationship between the craniomandibular system and sacroiliac articulation is unclear. In the current case, NT can be used in craniomandibular disorders that have pathogenic influence on the sacroiliac junction.	Pain assessed with VAS
Bölük Şenlikci H. et al.	2021	Randomized clinical trial	36	De Quervain´s tenosynovitis	20ml of procaine subcutaneous infiltration	Conventional treatment	NT was effective in reducing pain in patients with Tenosynovitis De Quervain	Pain evaluated with VAS and functionality evaluated with Duruöz Hand
Brobyn T.	2015	Case series	3	Chronic refractory pain	0.5% of procaine	No comparative group	Inference field-type neural therapy on scars can generate significant improvement and long-term pain control	Decreased pain intensity
Caponnetto V. et al.	2021	Systematic review	7 studies	Cervicogenic headache	Differences between studies: lidocaine, bupivacaine, alone or with steroid (methylprednisolone or dexamethasone)	No comparative group	Effective in reducing cervicogenic pain in acute or interictal phase, as well as being a relatively safe procedure.	Variability in outcome measurement pattern. Use of VAS or Numeral Rating Scale (NRS.). Frequency of pain and duration of pain attack

Caputi C. et al.	1997	Case report	27	Migraine	0,5 to 1 ml of bupivacaine 0,5% intradermic injection	No comparative group	Block of the greater occipital and supraorbital nerves appears to be effective in the treatment of migraine, but controlled studies are required.	Pain assessed with the total pain index (TP1)
Celia T. et al.	2016	Cohort study	168	Women with persistent pain after 10 days postpartum	1% procaine subdermal infiltration	No comparative group	The application of subdermal procaine is associated with the reduction of persistent postpartum pain	Degree of pain according to VAS, and number of treatment doses received
Corujeira M. et al	2010	Prospective study	21	Myofascial pain	1-2 ml 1% of lidocaine infiltration	Acupuncture	Positive effect of the infiltration in the decreased pain intensity	Decreased pain intensity
Couto C. et al.	2014	Randomized clinical trial	78	Myofascial pain	0.2 - 0.5 ml 1% of lidocaine infiltration at trigger points	Placebo and intramuscular stimulation	Intramuscular stimulation was better than placebo and lidocaine infiltration	Pain and depression assessed by scales
Cuadrado M. et al.	2017	Case series	18	Migraine	Bupivacaine 0,5%, 2 mL infiltration	No comparative group	Greater occipital nerve block seems effective in managing migraine symptoms with prolonged or persistent aura.	Pain assessed with VAS
Darabad R. et al.	2020	Case series	3	Cancer-related facial pain	1% lidocaine subcutaneous infiltration and 0.25% bupivacaine stellate ganglion infiltration	No comparative group	Stellate ganglion block is a therapeutic alternative in patients with facial pain associated with cancer or radiotherapy for cancer, and may improve quality of life in these patients	Numerical Rating Scale (NRS), use of analgesic medications
De las torres M. et al.	2008	Randomized clinical trial	60	Renal lithiasis	1.5ml 1% of lidocaine intradermic infiltration	No comparative group	Patients managed to expel the lithiasis from the fifth treatment session, regardless of the location and up to a diameter of 12mm	Expulsion from calculation

Díaz Alcorta T. et al.	2000	Non-randomized clinical trial	88	Alopecia areata	0.5% of procaine subcutaneous infiltration	Conventional treatment	Neural therapy proved to be a new effective therapeutic modality in the treatment of alopecia areata.	Repopulation of alopecic areas
Egli S. et al.	2015	Case series	280	Chronic refractory pain	Lidocaine and procaine intradermic infiltration	No comparative group	Long-term pain control with this therapy	Pain evaluation in a five-level scale
Eugene L. et al.	2017	Randomized clinical trial	120	Ulcerative colitis	10ml 1% lidocaine stellate ganglion block	Conventional treatment	Stellate ganglion block improves the efficacy of treatment in chronic ulcerative colitis, relieves clinical symptoms in patients and reduces the level of inflammatory factors.	Remission of symptoms and IL-8 values
Expósito Reyes O. et al.	2007	Phase II clinical trial	240	Cyclic breast dysplasia	2% of procaine diluted 1% with 0.9% saline subcutaneous and intradermic infiltration	Vitamin E (group II), with progesterone (group III) and with analgesics (group IV)	Satisfactory response with this treatment	Pain relief
Fern L. et al.	2020	Case series	2	Headache	Not described	No comparative group	Nerve block may be an optimal approach for the management of severe headache	Impact Test and exacerbation control
Fischer L. et al.	2015	Case report	1	Post-tonsillectomy pain	1% procaine infiltration in postsurgical scare	No comparative group	This is the first report of a successful therapeutic infiltration of a tonsillectomy scar in a patient refractory to medical treatment for several years.	Decreased pain intensity
Fisher BM. et al.	2019	Cohort study	477	Femur fracture	Local anesthetic	No comparative group	LA injection is associated with a reduction in opioid use in geriatric fracture patients with equivalent pain scores.	Amount of analgesia required measured in morphine equivalent milligrams (MME), days of hospital stay, and pain assessed with VAS

Fleckenstein J. et al.	2018	Case report	1	Chronic plantar fasciitis	< 1 ml 1% procaine	No comparative group	Effectiveness with this therapy allowing a prompt return to sports practice.	Pain assessed with VAS
Ga H. et al.	2007	Randomized clinical trial	40	Myofascial pain syndrome	0.2ml of 0.5% lidocaine trigger point infiltration	Acupuncture	No statically significant differences	Pain intensity scores
Ga H. et al.	2007	Randomized clinical trial	43	Myofascial pain syndrome	0.5 of lidocaine infiltration al trigger points	Intramuscular stimulation	No comparative improvement with de intramuscular stimulation	Pain intensity scores
Garvey T. et al.	1989	Randomized clinical trial	63	Low back pain	1% 1,5 ml of Lidocaine intradermic infiltration	Conventional treatment	The authors consider the injection of substances apparently not a critical factor and the direct mechanical stimulation of the trigger point seems to achieve equal symptomatic relief.	Average of pain reduction
Gibson RG. et al.	1999	A pilot study followed by a double-blind, placebo-controlled randomized study.	61	Multiple sclerosis	1% of lidocaine subcutaneous infiltration	Placebo	Positive effect at the disability evaluation	Improvement on disability scales
Goel V. et al.	2019	Systematic review	67 studies	Complications during the stellate ganglion nerve	No reported / local infiltration	No comparative group	Most reported complications of stellate ganglion block are during or immediately after the block. They are also reported with both technical and imaging guide blockages. It notes the importance of continuous monitoring and access to resuscitation equipment	Report of the different complications reported in the literature secondary to stellate ganglion block

Gonzalez-Rivas G. et al.	2018	Non-randomized clinical trial	178	Post-surgical pain due to mammoplasty	0,33% of procaine intradermic infiltration	Acupuncture	Despite the limitations of the study design, the use of NT may be recommended as an adjunctive treatment to reduce postoperative pain in patients undergoing axillary augmentation mammoplasty.	Post-surgical pain assessment
Gul H. et al.	2017	Randomized clinical trial	44	Chronic migraine	Bupivacaine 0,5% 1 ml intradermic injection	Placebo	Positive effect of the blockage	Days of headache and pain evaluated with VAS
Haller H. et al.	2018	Qualitative study	22	Chronic disease	25–30 15 of procaine infiltration into trigger points	No comparative group	Patients treated with procaine injections reported different psychophysiological outcomes that contributed to the understanding of the mechanisms underlying TN	Physical and emotional symptoms
Hasirci B. et al.	2022	Case series	53	Cervicogenic headache	Lidocaine 2% local infiltration	No comparative group	Repeated occipital nerve block was effective in reducing pain in patients with migraine and tension headache and may be an alternative treatment in patient's refractory to other treatments.	Pain intensity assessed with VAS
Hokenek N. et al.	2021	Randomized clinical trial	128	Acute migraine	0.75 ml de 1% lidocaine intradermic injection	Occipital Nerve Block (GON) Plus Supraorbital Nerve Block (SON) and Placebo	Supraorbital nerve block, greater occipital nerve block, and a combination of these two blocks are effective treatment methods in the acute treatment of migraine.	Pain assessed with VAS and Likert scale, requiring additional analgesic treatment

Hou J. et al.	2022	Before and after study	81	Migraine	6 ml ropivacaine 0,15%	No comparative group	Blocking is an effective method of treating migraine. With decreased pain and disability which could improve the quality of life.	Migraine Disability Rating Scale (MIDAS) and Numerical Rating Scale (NRS) at 3-month follow-up
Huang Y. et al.	2022	Case report	1	Unexplained chronic pain	ropivacaine 0.16% Stellate ganglion infiltration	No comparative group	Effective in improving chronic pain not medically explained, suggesting a clinical trial to evaluate the effectiveness of the treatment.	Pain intensity assessed with VAS, HAMA (Hamilton anxiety scale)
Hui F. et al.	1999	Case reports	56	Post herpetic neuralgia	1% of procaine infiltration in the affected dermatomes	Combined therapies: acupuncture, Chinese herbal therapy, meditation, or nerve block with LA.	Combined therapies are an effective treatment for postherpetic neuralgia	Average of pain reduction in postherpetic neuralgia (PHN)
Iguchi M. et al.	2002	Randomized controlled clinical trial	60	Renal pain	10-15 ml 15 lidocaine subcutaneous infiltration	Butylscopolamine	Safe and effective method to renal colic reduction	Pain reduction
Inan L. et al.	2015	Randomized clinical trial	84	Chronic migraine	1.5 ml 0.5% bupivacaine intradermic injection	Placebo	Results suggest that major occipital nerve block is superior in pain control in intensity and days compared to placebo	Days of headache and pain evaluated with VAS
Inoue M. et al.	2009	Randomized clinical trial	26	Chronic low back pain	5 mg of dibucaine hydrochloride infiltration	Acupuncture	Acupuncture has a more immediate effect and sustained effect on pain control	Pain assessment
Inoue M. et al.	2014	Randomized clinical trial	26	Chronic low back pain	Dibucaine 5mg/5ml intradermic injection	Conventional treatment	Both lidocaine injection and acupuncture relieve pain, but acupuncture was superior for immediate pain control and sustained effects	Pain assessed with VAS

István M. et al.	2018	Case series	12	Raynaud´s syndrome	5ml 0.25% of bupivacaine ganglion infiltration	No comparative group	More studies are needed	Skin temperature, trophism and pain assessed with VAS
Jeon Y. et al.	2015	Case report	1	Atypical facial pain	6 ml lidocaine 1,5% Intradermic injection	No comparative group	Case report documents pain relief after 2 months of follow-up	Pain control
Jonnavithula N. et al.	2019	Randomized clinical trial	30	Postoperative pain at the donor site in buccal mucosal graft urethroplasty	1 mL of 0.5% bupivacaine intradermic injection	No comparative group	Infraorbital nerve block is associated with postoperative analgesia and facilitates early food intake, mitigating donor site morbidity and providing satisfaction.	Intraoral morbidity and patient satisfaction
Julide O. et al.	2021	Case series	280	Chronic and refractory pain	Procaine and lidocaine intradermic infiltration	No comparative group	The low costs of LAs, the reduced number of consultations required, the reduced intake of analgesics and the lack of adverse effects also suggests the practicality and cost-effectiveness of this type of treatment. Controlled trials are required for assess the true effect of TN	Pain control
Kahokehr A. et al.	2011	Systematic review and meta-analysis	426	Abdominal pain due to laparoscopic surgery	Intraperitoneal anesthetics	Placebo	In the postsurgical period this therapy is effective in the management of abdominal pain and the use of opioids	Pain relief and use of opioids to pain management
Kamanli A. et al.	2005	Randomized controlled clinical trial	29	Myofascial pain syndrome	1 ml of 0.5% lidocaine infiltration	Botulinum toxin and dry injection into trigger points.	Positive effect	Improvement of symptoms
Karadaş Ö. et al.	2013	Randomized clinical trial	48	Chronic tension-type headache	0.5% of lidocaine	Placebo	This therapy may be and alternative for the headache pain relief	Pain evaluation by scales

Kim J. et al.	2017	Systematic review and meta-analysis	1,645 (9 studies)	Postherpetic neuralgia	Bupivacaine	Placebo and acyclovir	The use of blockages during the acute phase of herpes zoster shortens the duration of related pain and the incidence of postherpetic neuralgia	Incidence of postherpetic neuralgia, duration of acute pain
Kimura T. et al.	2005	Case report	7	Hypertension	Mepivacaine 1-2% 5 to 8 ml Intradermic injection	No comparative group	It is considered that the diffusion of LA can generate a vagal block. Monitoring of blood pressure figures is recommended in those who receive blockages	Hypertension and tachycardia
Kindberg S. et al.	2008	Randomized clinical trial	129	Pain after postpartum surgical repairs	4% of lidocaine intradermic infiltration	Acupuncture	Ear acupuncture was less effective for pain relief compared to a LA. No differences were observed in wound healing, need for wound revision or dyspareunia. Patient satisfaction with the assigned pain relief method was lower in the auricular acupuncture group.	Pain assessed with VAS
Korucu O. et al.	2018	Randomized clinical trial	60	Acute migraine	1 mL of 0.5% bupivacaine intradermic injection	Placebo and analgesics	Despite being an invasive procedure, a GON block could be an effective option. For the treatment of acute migraine in the emergency room due to its fast, easy, and safe application.	Pain scale score
Kouri M. et al.	2021	Case report	17	Chronic headache	0.3 mL 0.5% bupivacaine injection into each nostril	No comparative group	Sphenopalatine ganglion blockade could have a beneficial effect at the level of headache prevention in adolescents, with impact	Patient's Global Impression of Change score y Revised Children's Anxiety and Depression Scale (RCADS)

							on PGIC and the RCADS depression subscale.	
Kronenberg R. et al.	2018	Case report	1	Chronic pelvic pain syndrome	1% 5ml of procaine vesicoprostatic plexus infiltration	No comparative group	This is the first report of successful therapeutic infiltration of the vesicoprostatic plexus using a LA.	Pain assessment
Lee A. et al.	2018	Case series	3	Postoperative hiccups	0.5ml 0.2% ropivacaine stellate ganglion infiltration	No comparative group	Stellate ganglion block is an effective method for controlling hiccups	Hiccup
León R. et al.	2016	Case series	9	Chronic pelvic pain	Procaine intradermic infiltration	No comparative group	NT is an alternative for the treatment of patients suffering from chronic pelvic pain, since it allowed a noticeable decrease in pain, by producing pain modifications in VAS and a decrease in the frequency of pain per week.	Pain assessed with VAS
Levene R.	2015	Cohort study	44	Chronic low back pain	Local anesthetic infiltration at trigger points	Physiotherapy and psychotherapy as a multidisciplinary assessment	Multidisciplinary assessment of low back pain has a positive effect	Pain and depression evaluation by scales
Liu Q. et al.	2020	Case report	1	Post-stroke pain	2 ml lidocaine 2% Ultrasound-guided intradermal injection	No comparative group	Stellate ganglion block in a treatment option in pain refractory to pharmacotherapy	VAS-assessed pain, Barthel Activities of Daily Living Scale
Loriz O.	2013	Cohort study	46	Painful shoulder	Segmental anesthetic	No comparative group	This therapy is useful to pain relief and use of oral medication in this pathology	Pain evaluation by scales

Lóriz O.	2015	Non-randomized clinical trial	58	Osteoarthritis of the knee	1% of procaine infiltration	Conventional treatment	This therapy may be a good therapy for knee pain relief	Pain evaluation by scales
Lóriz O. et al.	2011	Before and after study	82	Acute and chronic pain	1% of procaine intradermic infiltration	No comparative group	Effectiveness with this therapy in the decreased pain intensity and oral medication	Assessment of pain and oral medication
Lynch JH. et al.	2016	Case series	30	Post-traumatic stress syndrome	7 mL 0.5% ropivacaine stellate ganglion block	No comparative group	As a result of the significant reduction in observed hyperarousal and avoidance symptoms, this study supports the incorporation of stellate ganglion block into post-traumatic stress syndrome treatment plans.	PTSD (post-traumatic stress disorder) Checklist-Military. (PCL-M)
M Pfister. et al.	2009	Case series	2	Complex regional pain syndrome	4 ml of procaine	No comparative group	LA infiltration plus oral medication is effective in this pathology	Pain control
Machado F. et al.	2021	Case series	6	Headache after SARS-CoV-2	2% lidocaine intranodal blockade	No comparative group	This study has several limitations, but it is the first report to show the efficacy of intranodal blockade in headache control.	Pain control
Maki P. et al.	2016	Randomized clinical trial	36	Learning and verbal memory	0.5% bupivacaine (5ml) fluoroscopy-guided intranodal infiltration	Placebo	This pilot trial provides new tentative evidence of the impact of blocking on verbal memory with no impact on logical memory	Verbal memory assessed with California Verbal Learning Test (CVLT) and logical memory assessed with Logical Memory Subtest of Wechsler Memory Scale-Revised

McCormick Z. et al.	2018	Randomized clinical trial	4	Postamputation pain	1-2 ml lidocaine 1% Fluoroscopy-guided injection	Placebo	Study with few patients, little power, without documentation of the physiopathological mechanism that impacts the outcome. Further studies required	Residual phantom limb pain, pain and quality of life
Medrano G. et al.	2011	Open, multicentric clinic trial	34	Low back pain	1% of procaine subcutaneous infiltration	No comparative group	Positive effect in the pain reduction with this therapy	Pain evaluation by scales
Mellick LB. et al.	2008	Retrospective cohort study	114	Orofacial pain	0.5% Bupivacaine local infiltration	No comparative group	therapeutic response was documented in 94% of patients. Complete pain relief in 48%, clinical improvement in 18% and partial relief in 28% of the 114 documented patients.	Pain relief
Mellick LB. et al.	2010	Retrospective cohort study	13	Headache	1 to 1,5 ml 0.5% of bupivacaine	No comparative group	Partial and complete improvement of headache was demonstrated	Pain relief
Mermod J. et al.	2008	Cohort study	405	Musculoskeletal diseases	No intervention	Conventional treatment	More satisfaction with neural therapy than with conventional treatment	Neural therapy satisfaction
Michels T. et al.	2007	Retrospective cohort study	238	Cervical pain	0.2 ml of 0.5% of Mepivacaine intradermic infiltration	No comparative group with alternative therapies or placebo	Long-term remission of the symptoms	Remission of the symptoms
Michels T. et al.	2018	Cohort study	127	Peripheral pain	0.1 a 0.2 ml 0.5% of mepivacaine	No comparative group	Local anesthetics in low concentrations and small amounts proved to be highly effective in the treatment of peripheral pain.	Pain assessed with VAS
Millan M. et al.	1991	Randomized clinical trial	30	Myofascial pain	1% of procaine intradermic infiltration	Placebo	It is recognized that the therapeutic value of procaine in the management of	Pain threshold and intensity

							myofascial pain is questionable.	
Mitidieri AMS. et al.	2020	Randomized clinical trial	35	Abdominal myofascial pain syndrome	2ml 1% of lidocaine	Ashi acupuncture	No statically significant differences between de two therapies.	Pain assessed with VAS
Molnár I. et al.	2019	Case series	16	Pain due to unresectable pancreas cancer	5ml 1% of lidocaine retrocrural and guided puncture under fluoroscope	No comparative group	What was observed in the case series shows that celiac plexus block could be a complementary treatment to unresectable pancreas cancer, with improvement in quality of life	Quality of life assessed with SF36, and pain control assessed with VAS 35 days after intervention
Moon S. et al.	2020	Case report	2	Chronic headache	5 ml lidocaine 1,5% intradermic injection	No comparative group	The authors consider the block of the stellate ganglion could be to improve headache and disability in patients with chronic migraine	Pain evaluated with VAS and MIDAS
Naja Z. et al.	2009	Randomized clinical trial	47	Postdural puncture headache	Lidocaine al 2%, 3 ml intradermic injection	Hydration, rest an analgesic	Findings suggest occipital nerve block may be a successful alternative Treatment for patients suffering from post-puncture headache with an earlier return to regular activities	Pain assessed with VAS
Nassar A. et al.	2021	Before and after study	93	Headache	5ml 1% of lidocaine intramuscular and subcutaneous infiltration	No comparative group	Differences were found in 7 of the 8 domains of SF 36 when comparing before and after and improvement in the frequency of symptoms	Quality of life assessed with SF 36 and frequency of symptoms
Okmen K. et al.	2016	Case series	60	Migraine	bupivacaine 0,5% local infiltration	No comparative group	Although infiltration of the greater occipital nerve does not cure migraine, it is useful as a support treatment. It can	EVA, MIDAS (The Migraine Disability Assessment Questionnaire) al 3er y 6o mes

							be used in different age groups.	
Ong J. et al.	2014	Systematic review and meta-analysis	266	Myofascial pain	2 ml 1% of Lidocaine	Dry injection into trigger points	No significant differences between these interventions	Pain evaluation by scales
Özer D. et al.	2019	Randomized clinical trial	87	Migraine	2 ml lidocaine 1% intradermic injection	Placebo	greater occipital and supraoccipital nerve block was more effective than placebo	Days of headache and pain evaluated with VAS
P. Barbagli.	1998	Cohort study	115	Knee pain	0.5 to 1 ml 0.5 - 1% of lidocaine intradermic infiltration	No comparative group	Reported results suggest good treatment effectiveness for knee pain control, but randomized clinical trials are required	Pain assessed at 1-, 3- and 6-months follow-up (percentage of subjective improvement)
Paneque R. et al.	2009	Cohort study	50	Acute alveolar dental abscess	No reported	No comparative group	Study with important limitations, which reports in general the symptomatic improvement	Pain and inflammation of root canals
Peraza R. et al.	2011	Open phase II clinical trial	47	Epicondylitis	0.2–0.5 ml 2% of procaine infiltration at trigger points	No comparative group	Significant decrease in pain and functional improvement	Pain and functional assessment
Perloff M. et al.	2018	Case report	9	Refractory trigeminal neuralgia	0,5 ml bupivacaine 0,25% intradermic injection	No comparative group	patients achieved rapid and sustained pain relief with the blockage.	Percentage of pain control
R Spernol. et al.	1982	Case reports	10	Neuropathic disorders of the bladder	Paraurethral injection of 2.5ml of 1% lidocaine solution	No comparative group	Effective treatment in motor and sensory urgency	Improvement of the symptoms of urgency and frequency and side effects
Rae O. et al.	2020	Randomized clinical trial	113	Post-traumatic stress syndrome	7 to 10 mL 0.5% ropivacaine stellate ganglion block	Placebo	The findings suggest the possible effect of stellate ganglion block on the management of post-traumatic stress, so further studies are required	Clinician-Administered PTSD Scale for DSM-5 (CAPS-5)

Reis M. et al.	1990		16	Postherpetic neuralgia	15ml 1% lidocaine local infiltration	No comparative group	Variable pain control with the use of block, which showed greater effect in the absence of associated psychological symptoms	Pain reduction
Rey Novoa M. et al.	2021	Case series	5	Vulvodynia	0.5% of procaine	No comparative group	Procaine injection showed favorable outcomes, further studies required	Pain assessed with VAS, frequency of symptom improvement
Reyes-Corona L. et al.	2009	Prospective cohort study	72	Pelvic inflammatory disease	1% of Procaine or lidocaine intradermic infiltration	No comparative group	Efficacy of this therapy in this pathology	Resolution of symptoms as good, regular, or bad response
Ruiz M. et al.	2016	Case series	60	Migraine	1:1 bupivacaine 0.5% y mepivacaine 2%	No comparative group	Anesthetic block of pericranial nerves using sensitivity to Palpation as a selection criterion is a safe and potentially effective procedure as a preventive treatment of migraine	Percentage response in pain control, adverse events
Ruiz-Mejía A. et al.	2020	Case report	1	Acute stress disorder	Procaine trigger points infiltration	No comparative group	The neuraltherapeutic approach allowed, on the one hand, to identify a link between the patient's dissociative symptoms and the associated organic alteration, and, on the other, to make an intervention with which the resolution of such symptoms was achieved.	Pain relief
Russi Garzón. J	2012	Before and after study	64	Musculoskeletal pain	0.5% of procaine	No comparative group	The use of this therapy has a positive impact in the quality of life	Quality of life assessed with SF-36
Saha FJ. et al.	2014	Case report	1	Chronic pain syndrome	2ml of procaine 1%	No applicable	This therapy may be effective in complicated cases of vulvodynia	Pain evaluation by scales

Sencan S. et al.	2022	Randomized clinical trial	34	Chronic coccygodynia	3 ml d prilocaine 2% Fluoroscopy-guided injection	Caudal steroid injection	The two interventions evaluated showed usefulness in the treatment of chronic coccydynia	Pain intensity. Presence of neuropathic pain
Sermeus L. et al.	2018	Randomized clinical trial	20	Pain due to shoulder arthroscopy	Levobupivacaine 0.5% stellate ganglion block	Paravertebral cervical block	No additional antinociceptive benefit of adding a stellate ganglion block to cervical paravertebral block was demonstrated for the treatment of acute pain caused by arthroscopic surgery.	Nociceptive and nocisensitive thresholds
Sevgi Gümüş Atalay. et al.	2020	Randomized clinical trial	92	Lateral epicondylitis	0.5ml 2% Lidocaine subcutaneous infiltration	Extracorporeal shockwave therapy	No statically significant differences between de two therapies.	Pain assessed with VAS and functional recovery assessed with DHI score
Tamam Y. et al.	2017	Cohort study	28	Multiple sclerosis	40 ml of lidocaine Intradermic injection into the urogenital segment	No comparative group	Neural therapy could be effective in the treatment of neurogenic bladder in patients with multiple sclerosis, more studies are needed	Control of neurogenic hyperactivity of the detrusor with urodynamic evaluation and quality of life evaluated with MSQL-54
Tang Y. et al.	2017	Systematic review and meta-analysis	297 (6 studies)	Migraine	Lidocaine or bupivacaine or triamcinolone, triamcinolone+Lidocaine 2%, o Triamcinolone + Bupivacaine 0,5% local infiltration	Placebo	The use of major occipital nerve block in migraine patients is recommended	Severity of pain, number of days with pain, consumption of analgesic medications
Tepe N. et al.	2020	Cohort study	82	Chronic medication overuse headache	3 mL 0.25% bupivacaine intradermic injection	nerve block plus supraorbital nerve block	The study showed significant reductions in headache parameters in both groups. However, pain score, analgesic intake, number of painful days and duration of pain improved	Pain assessed with symptom score, number of days with pain in 1 month

							significantly in the combined blocks group.	
Terzi T. et al.	2002	Randomized clinical trial	60	Migraine, tension-type headache and cervicogenic headache	1ml prilocaine 2% intradermic injection	Placebo	Greater occipital nerve block is effective and useful for the diagnosis of cervicogenic headache	Headache evaluated with VAS
Trevin G. et al.	2012	Phase IIb clinical trial	70	Acute mechanical low back pain	Procaine infiltration	No comparative group	Positive effect with this therapy	Effectiveness of neural therapy
Triantafyllidi H. et al.	2016	Cohort study	22	Hypertension	2% lidocaine on cotton applicator	No comparative group	Sphenopalatine ganglion block could be a promising, non-invasive, safe, painless, and easy-to-perform blood pressure lowering therapeutic option	Control of blood pressure, percentage of responders (defined as reduction of 5mmHg at 24h after blockage)
Triantafyllidi H. et al.	2018	Randomized clinical trial	44	Hypertension	Bilateral cervical injection of 2% lidocaine	Placebo	Bilateral sphenopalatine block is a minimally invasive option, which decreases the values of arterial tension	Reduction of blood pressure values, percentage of responders defined with post-blockade reduction of systolic blood pressure (SBP) more than 5mmHg
Ural F. et al.	2017	Randomized clinical trial	42	Epicondylitis	16ml 0.5% of lidocaine intradermic infiltration	Non-invasive treatment	NT improves pain and functional status in patients with lateral epicondylitis	Pain assessed with VAS; hand performance status assessed with Duruoz hand index (DHI)
Valencia Moya A. et al.	2020	Randomized clinical trial	55	Chronic low back pain	20mg of procaine	Paravertebral lumbar infiltration, intramuscular injection two centimeters from the spinous processes of L3-L4.	Treatment with TNS achieved statistically significant pain relief at three months. A statistically significant reduction in disability associated with low back pain was observed in the group treated with	Pain assessed with VAS at week 0, 3 and 12 months

							combined injection at 3 months, not being significant when comparing the groups with each other.	
Vanterpool S. et al.	2020	Case series	50	Occipital headache	Bupivacaine 0.25%, by alternative suboccipital approach with paresthesia technique	No comparative group	The alternative suboccipital technique with paresthesia for occipital nerve block is an option in the treatment of neuropathic occipital headache.	Rating Scale (NRS)
Villamizar Olarte D. et al.	2016	Randomized clinical trial	66	Neck or low back pain	0,2-0,4 cc 1% of procaine intradermic injection	Placebo	NT has been shown to be effective and safe in the treatment of neck and low back pain, as well as improving the function of patients with low back pain and those treated in interfering fields	VAS-assessed pain, functional disability, medication use
Walega D. et al.	2014	Randomized clinical trial	40	Vasomotor symptoms in women with natural or surgical menopause	5ml 0.5% bupivacaine stellate ganglion block	Placebo	Stellate ganglion block may provide effective treatment of vasomotor symptoms in women seeking non-hormonal treatments due to safety concerns and personal preference	Vasomotor symptoms
Wang S. et al.	2017	Case report	1	Epilepsy	5 ml 1% lidocaine stellate ganglion infiltration	No comparative group	Stellate ganglion block may be an effective treatment of intractable partial epilepsy. Further studies are required to verify validity.	Seizures
Weinschenk S. et al.	2013	Case report	1	Vulvodynia	3-15ml 1% of procaine	No comparative group	This therapy may be useful in complicated vulvodynia	Adverse effects and pain control

Weinschenk S. et al.	2016	Cohort study	17	Neck pain refractory to therapy	0.5 ml 1% of procaine Injection into the palatine veil	No comparative group	Patients with chronic pharyngeal disease may benefit from neural therapy	Pain assessed with an index of three levels (0,1,2)
Weinschenk S. et al.	2016	Case series	20	Vulvodynia	1% procaine infiltration	No comparative group	Perineal pudendal injection is an effective and safe method for anesthesia in diagnostic processes (vulvar biopsy) and therapeutic indications (pudendal neuralgia), and regional anesthesia in perinatal settings.	Analgesia and adverse events
Weinschenk S. et al.	2017	Randomized clinical trial	177	Allergic reaction evaluation	Procaine 1%, lidocaine 0.5%, mepivacaine 1%	Placebo	The study showed no allergenicity of LAs. Procaine injection induces an erythema that persists for more than 10 minutes and is probably caused by vasodilation, rather than an allergic reaction.	Type 1 allergy
Yavuz F. et al.	2016	Case report	1	Paralysis of the facial nerve	10 ml 0.4% of lidocaine subcutaneous infiltration	No comparative group	Neural therapy could be effective in the treatment of facial paralysis	Response to therapy with House-Brackmann score
Yera-Nadal J. et al.	2003	Prospective cohort study	57	Herpes Zoster	Trigger point injection of 0.5% of Procaine	N comparative group	Positive effect in the neuralgia pain reduction	Pain reduction assessment
Yılmaz E. et al.	2021	Randomized clinical	50	Chronic low back pain	2ml 0,4% of lidocaine	4ml of lidocaine 2% combined with betamethasone	NT may be an alternative for the management of chronic low back pain that does not respond to conventional treatment	Pain assessed with VAS, and disability assessed with the Roland Morris Disability Questionnaire (RMDQ)

Yilmaz V. et al.	2019	Case series	20	Migraine	Bupivacaine 0,25%, 2mL local infiltration	No comparative group	Major occipital nerve block reduces the severity of pain in patients with migraine and fibromyalgia and improves function.	VAS-assessed pain, Fibromyalgia Impact Questionnaire (FIQR) function and MIDAS (Migraine Disability assessment test)
Zagorulko O. et al.	2019	Cohort study	43	Cervicogenic headache	Articaine 2% intradermic injection	Lidocaine 2%	Occipital nerve blocks with 2% articaine show a significant decrease in pain intensity and duration of pain paroxysms in a short period of time for patients with cervicogenic headache.	VAS-assessed pain, individual duration of paroxysmal pain
Zarate MA. et al.	2020	Randomized clinical trial (pragmatic)	29	Temporomandibular dysfunction	0.2% of lidocaine	Dextrose prolotherapy (DPT)	Intra-articular DPT resulted in clinically important and statistically significant improvement in pain and dysfunction at 12 months compared with lidocaine injection.	Intensity of temporomandibular pain and mandibular dysfunction (numerical scale of 0-10) dysfunction at 3 and 12 months
Zhang H. et al.	2018	Systematic review and meta-analysis	7 studies	Migraine	Lidocaine, bupivacaine intradermic injection	Placebo	Major occipital nerve block significantly decreases pain intensity, with an adequate safety profile without an increase in adverse events	Pain intensity, headache duration, adverse effects
Zhu Z. et al.	2019	Cohort study	132	Pain due to laparoscopic radical gastrectomy	40ml 0.5% of Ropivacaine	No analgesia	Infiltration with ropivacaine showed an analgesic effect in the postoperative period, with a good safety profile, randomized clinical trials are required to confirm the findings	Pain intensity assessed on a numerical scale, need for rescue medication and complications

Vinyes D, Muñoz-Sellart M, Fischer L. Therapeutic Use of Low-Dose Local Anesthetics in Pain, Inflammation, and Other Clinical Conditions: A Systematic Scoping Review. *Journal of Clinical Medicine*. 2023; 12(23):7221. https://doi.org/10.3390/jcm12237221
